# Comparison of *NTRK* fusion detection methods in microsatellite-instability-high metastatic colorectal cancer

**DOI:** 10.1007/s00428-023-03538-1

**Published:** 2023-04-17

**Authors:** Suzanna J. Schraa, Ellen Stelloo, Miangela M. Laclé, Joost F. Swennenhuis, Lodewijk A. A. Brosens, Remond J. A. Fijneman, Harma Feitsma, Miriam Koopman, Wendy W. de Leng, Geraldine R. Vink, Guus M. Bol

**Affiliations:** 1 Department of Medical Oncology, University Medical Center Utrecht, Utrecht University, Utrecht, Netherlands; 2 Cergentis BV, Utrecht, Netherlands; 3 Department of Pathology, University Medical Center Utrecht, Utrecht University, Utrecht, Netherlands; 4 Department of Pathology, Netherlands Cancer Institute, Amsterdam, Netherlands; 5 Department of Research and Development, Netherlands Comprehensive Cancer Organisation (IKNL), Utrecht, Netherlands

**Keywords:** *NTRK* fusions, Fusion detection, Immunohistochemistry, Sequencing, FISH

## Abstract

Tropomyosin receptor kinase (TRK) inhibitors have been approved for metastatic solid tumors harboring *NTRK* fusions, but the detection of *NTRK* fusions is challenging. International guidelines recommend pan-TRK immunohistochemistry (IHC) screening followed by next generation sequencing (NGS) in tumor types with low prevalence of *NTRK* fusions, including metastatic colorectal cancer (mCRC). RNA-based NGS is preferred, but is expensive, time-consuming, and extracting good-quality RNA from FFPE tissue is challenging. Alternatives in daily clinical practice are warranted. We assessed the diagnostic performance of RNA-NGS, FFPE-targeted locus capture (FFPE-TLC), fluorescence in situ hybridization (FISH), and the 5′/3′ imbalance quantitative RT-PCR (qRT-PCR) after IHC screening in 268 patients with microsatellite-instability-high mCRC, the subgroup in which *NTRK* fusions are most prevalent (1–5%). A consensus result was determined after review of all assay results. In 16 IHC positive tumors, 10 *NTRK* fusions were detected. In 33 IHC negative samples, no additional transcribed *NTRK* fusions were found, underscoring the high sensitivity of IHC. Sensitivity of RNA-NGS, FFPE-TLC, FISH, and qRT-PCR was 90%, 90%, 78%, and 100%, respectively. Specificity was 100% for all assays. Robustness, defined as the percentage of samples that provided an interpretable result in the first run, was 100% for FFPE-TLC, yet more limited for RNA-NGS (85%), FISH (70%), and qRT-PCR (70%). Overall, we do not recommend FISH for the detection of *NTRK* fusions in mCRC due to its low sensitivity and limited robustness. We conclude that RNA-NGS, FFPE-TLC, and qRT-PCR are appropriate assays for *NTRK* fusion detection, after enrichment with pan-TRK IHC, in routine clinical practice.

**Supplementary Information:**

The online version contains supplementary material available at 10.1007/s00428-023-03538-1.

## Introduction

Tropomyosin receptor kinase (TRK) inhibitors show durable responses in all tumor types harboring an *NTRK* fusion and are now a standard treatment option [1, 2]. Although *NTRK* fusions have been identified in all tumor types, their prevalence is typically low, complicating fusion detection in daily clinical practice. For instance, in metastatic colorectal cancer (mCRC), the prevalence of *NTRK* fusions is only 0.3% [3]. The vast majority of *NTRK* fusions are found in the subgroup of microsatellite-instability-high (MSI-H)/mismatch-repair-deficient (dMMR) tumors in which the prevalence is estimated to be 1–5% [4–6].

Implementation of *NTRK* fusion detection in routine care is hampered by the complexity of available testing strategies. International guidelines from both ASCO and ESMO recommend to use immunohistochemistry (IHC) as a screening assay followed by RNA-based next-generation sequencing (NGS) confirmation, which is considered the most sensitive test [7, 8]. However, RNA-based NGS is expensive, time-consuming, and may have decreased robustness. Specifically, extracting RNA from formalin-fixed, paraffin-embedded (FFPE) tissue is challenging due to crosslinking and fragmentation which decreases RNA quantity and quality [9, 10].

Ideally, molecular tests are implemented that are accurate, affordable, fast, and can be performed on archived FFPE tissue [11]. Alternative available tests for the confirmation of *NTRK* fusions include DNA-based NGS and fluorescence in situ hybridization (FISH) [12, 13]. More recently, the 5′/3′ imbalance quantitative RT-PCR (qRT-PCR) and FFPE-targeted locus capture (FFPE-TLC) have been added to the repertoire of gene fusion detection methods [14, 15]. Each assay has advantages and disadvantages, but head-to-head comparisons are lacking.

Like RNA-based NGS, DNA-based NGS can be used for multiplex and high-throughput analysis of gene fusions. DNA-based NGS has lower failure rates because DNA is more stable than RNA and is better preserved in FFPE tissues. However, the sensitivity for gene fusion detection of DNA-based NGS is limited by the large intronic regions with repetitive sequences, which are not covered by widely used targeted panels such as MSK-IMPACT and FoundationOneCDx [13]. FFPE-TLC, a novel DNA-based targeted NGS, can overcome this limitation of the standard DNA-based NGS. FFPE-TLC uses proximity ligation to obtain broad coverage on the fusion partners to detect fusion genes independent of the identification of the fusion breakpoint sequence. Recently, FFPE-TLC showed excellent diagnostic accuracy compared to FISH and DNA-NGS in detecting rearrangements in lymphoma FFPE samples [15].

FISH has a short turnaround time and is relatively inexpensive [16]. However, the fusion partner remains unknown, a positive test does not prove overexpression of the fusion product, and separate assays are needed to assess for rearrangements in *NTRK1*, *NTRK2*, and *NTRK3*.

The 5′/3′ imbalance quantitative RT-PCR (qRT-PCR), also known as Idylla GeneFusion Assay, combines reverse transcription quantitative real-time PCR (qRT-PCR) for the detection of known fusions and expression imbalance to detect fusions with unknown fusion partners, including *NTRK.* When a fusion is present, there will be an imbalance of the mRNA expression of the 3′ kinase domain, which is expressed because of its association with the novel fusion partner, and the original 5′ region which is not [14, 17]. This assay has a turnaround time of only three hours, is fully automated, and proves overexpression of the fusion protein. However, the fusion partner remains unknown and there is limited comprehensible quality or quantity control, while qRT-PCR faces the same challenge of using reduced quality RNA from FFPE tissue as RNA-NGS.

In order to find reliable alternatives for the detection of *NTRK* fusions that fits the need in routine care, we compare the diagnostic performance, defined as the robustness, sensitivity, and specificity, of RNA-based NGS, FFPE-TLC, FISH, and the qRT-PCR for the detection of *NTRK* fusions after pan-TRK IHC screening in MSI-H/dMMR mCRC.

## Materials and methods

### Patient selection and FFPE collection

All patients diagnosed with MSI-H/dMMR mCRC between 2015 and 2021 in the Netherlands were selected using the Netherlands Cancer Registry. All available formalin-fixed, paraffin-embedded (FFPE) tumor tissue blocks were retrieved using the Nationwide Network and Registry of Histo- and Cytopathology in the Netherlands (PALGA). Whenever possible, tissue of the primary tumor resection was used. If not available, tissue from a primary tumor biopsy or metastasis was used.

### Immunohistochemistry screening

We used pan-TRK IHC as a screening test. The received tissue blocks were cut into 4-µm-thick slides and stained using a Ventana bench mark ultra autostainer using antigen retrieval for 24 min with CC1 (EDTA) and 32 min antibody exposure with a rabbit pan-TRK monoclonal antibody (mAb) (clone EPR17341, Abcam, Cambridge, MA) in a dilution of 1:500 according to standard procedures. Brain tissue was used as positive control.

**Fig. 1:**
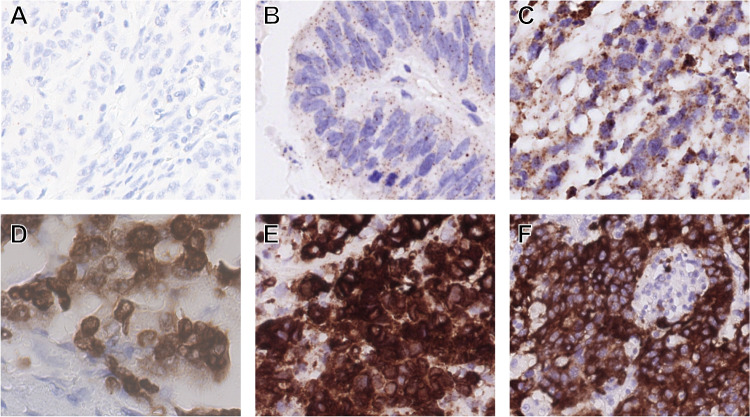
Pan-Trk IHC, 40 × original magnification. (**A**) Negative staining, no additional testing was performed. (**B**) Weak, non-specific granular cytoplasmic staining (scored negative), no *NTRK* fusion was found. (**C**) Moderate cytoplasmic staining, no *NTRK* fusion was found. (**D**) Strong cytoplasmic + nuclear staining, *LMNA::NTRK1* fusion was detected. (**E**) Strong cytoplasmic + perinuclear staining, *LMNA::NTRK1* fusion was detected. (**F**) Strong cytoplasm + membranous staining, *TPM3::NTRK1* was detected

All slides were examined by two independent qualified pathologists (MML and LAAB) and scored for percentage of positive tumor cells, intensity, and staining pattern (cytoplasmic, nuclear, perinuclear, and/or membranous). In case of discordancy between the pathologists, the highest intensity score was used underlining the use as screening method. Positive staining was defined as ≥ 1% of tumor cells showing staining above background in any pattern and any intensity. Tumors that showed weak, non-specific granular cytoplasmic staining (Fig. 1B) were scored negative. All tissue samples with positive staining and 11 negative samples with weak, non-specific staining were included in the comparative analysis and analyzed with RNA-NGS, FFPE-TLC, FISH, and qRT-PCR.

In addition, a subset of pan-TRK negative cases was selected to examine the sensitivity of pan-TRK IHC. For this analysis, we selected the subgroup with the highest prevalence of *NTRK* fusions (MSI-H/dMMR, *BRAF* wild-type, and *RAS* wild-type) for FFPE-TLC analysis. Previously, we reported a *NTRK* fusion prevalence of 22% in this subgroup [18]. Transcription of *NTRK* fusions detected by FFPE-TLC was confirmed by RNA-NGS.

### RNA-NGS

The Archer Fusionplex® Lung panel (ArcherDX, San Diego, USA) was used to assess *NTRK1*, *NTRK2*, and *NTRK3* rearrangements (Supplementary Table S1). RNA was isolated using the Maxwell® RSC 48 instrument and the Maxwell® RSC RNA FFPE kit (Promega, Madison, USA) from four FFPE sections of 10 µm with a minimum of 10% tumor cell content. Isolated RNA was quantified using the Quantus™ Fluorometer and the QuantiFluor®RNA system (Promega, Madison, USA). A quantitative PCR (qPCR)-based method was used to determine the quality of the mRNA in each sample prior to the targeted library preparation. When a sample did not meet the quality criteria (Cq quantitative PCR should be < 30 cycles), the isolation and quality control was repeated using slides from a different tumor tissue block (if available). Libraries were prepared using the Archer FusionPlex reagent kit for Illumina (ArcherDX, San Diego, USA) according to manufacturer’s instructions. Results were analyzed on the ArcherDX website (https://analysis.archerdx.com/), version 6.2 or 7.0. Criteria for analysis were minimum of 200.00 RNA reads, unique RNA start sites per GSP2 control ≥ 10, on target deduplication ratio ≥ 3 and ≤ 10. Fusions must have > 3 SS unique start sites, > 5 breakpoint spanning reads, 10% fusion reads, and must be in frame. All results were examined by the same molecular biologist (WWL), who was blinded for the IHC result.

### FFPE-targeted locus capture

A detailed description of FFPE-TLC was published before [15]. In brief, two to four FFPE sections of 10-µm thickness were deparaffinized to enable in situ DNA digestion by a restriction enzyme. After in situ ligation and overnight reverse crosslinking, standard protocols for library preparation and hybridization capture-based target enrichment were followed. Resulting libraries were paired-end sequenced on an Illumina platform. Two different capture panels were used to target *NTRK1*, *NTRK2*, *NTRK3*, and several other genes, including *BRAF*. For a subset of samples *KRAS*, *NRAS*, *EGFR*, and *PIK3CA* were also covered (Supplementary Table S2). One million on-target reads were aimed for 1 Mb region of interest.

After alignment of the raw sequencing data to the human genome, a computational pipeline PLIER was run to automatically detect *NTRK1*, *NTRK2*, and *NTRK3* gene fusions. In brief, PLIER detects genomic intervals with significantly increased coverage of proximity ligation products per target locus and calculates a *z*-score by comparing its observed proximity score to the related expected proximity score. This PLIER pipeline has been optimized based on lymphoma cases that nearly always present as interchromosomal (trans) fusions, with fusion partners on a different chromosome. For CRC, several intrachromosomal (cis) fusions have been described, like for example *LMNA*::*NTRK1*, with both genes being located on chromosome 1. Therefore, visual inspection was performed in addition to PLIER to detect these intrachromosomal rearrangements. The scientists (ES, JFS, HF) involved in rearrangement calling were blinded for the results of the other assays.

### Fluorescence in situ hybridization

FISH was performed on all pan-TRK positive cases [19]. *NTRK1*, *NTRK2*, and *NTRK3* break-apart FISH probes (Z-2167, Z-2205, and Z-2206, ZytoVision GmbH, Bremerhaven, Germany) were used in a stepwise approach. First, the *NTRK1* break-apart probe was used, as the *NTRK1* gene is the most common *NTRK* fusion detected in colorectal cancer. Negative cases were subsequently analyzed using the *NTRK3* break-apart probe. Lastly, FISH using the *NTRK2* probe was performed for cases that were negative for both the *NTRK1* and *NTRK3* FISH.

For all tumor samples, a minimum of 100 non-overlapping nuclei were scored for their signals. FISH was considered positive for an *NTRK* fusion if ≥ 10% tumor cells showed a separation of red and green signals with a minimum of two signal diameters. With respect to single red signals: FISH was considered positive if ≥ 15% tumor cells showed a single red signal and equivocal if 10–15% of tumor cells showed a single red signal. FISH analysis was performed by qualified analysts and interpreted by a molecular biologist (WWL).

### 5′/3′ imbalance quantitative RT-PCR (qRT-PCR)

For the qRT-PCR we have used the Idylla GeneFusion Assay (Biocartis NV, Mechelen, Belgium). One 10-µm-thick slide was used according to the manufacturer’s instructions. A result was obtained for *NTRK1*, *NTRK2*, and *NTRK3* individually. In case of errors or invalid test results, the assay was repeated once with another slide from the same tissue block. An invalid result for one out of three *NTRK* genes in the absence of a detected *NTRK* fusion was scored as invalid.

### Comparison between assays

For all assays, the robustness was determined. Robustness was defined as the percentage of analyzed samples that provided an interpretable result in the first run. The assay results were all reviewed by two independent molecular biologists (WWL and RJAF), and their consensus decision regarding the presence of *NTRK* fusions per sample was used as truth set reference. Using this truth set reference, sensitivity and specificity were calculated for RNA-NGS, FFPE-TLC, FISH, and qRT-PCR. Subsequently, detected mutations in *BRAF*^*V600E*^ and *KRAS* by RNA-NGS and FFPE-TLC were compared, and the level of agreement was evaluated.

### Statistical analysis

Statistical analysis was performed using R Statistical Software (v4.0.1) and the epiR package. *P*-values of < 0.05 were considered statistically significant.

## Results

We identified 383 patients diagnosed with MSI-H/dMMR mCRC between 2015 and 2021 in the Netherlands. Tumor tissue from 306 patients was collected, and 268 patients were included for the current analysis. The dropouts were due to administrative reasons (*n* = 77) or FFPE material did not contain sufficient cancer cells (*n* = 38) (Supplementary Fig. S1). Pan-TRK IHC was scored positive in 16 out of 268 (6%) patients (Fig. 1). All positive samples showed cytoplasmic staining and nine samples also showed nuclear, perinuclear, or membranous TRK expression (Supplementary Table S3). In general, there was homogenous pan-TRK staining with a median of 95% positive stained tumor cells (range: 10–100%) (Supplementary Table S3). Pan-TRK IHC negative samples often showed some weak, non-specific granular cytoplasmic staining (Fig. 1B). Patient characteristics of the *NTRK* positive patients have been published before [18]. No prior *NTRK* fusion tests were performed.

### Robustness of assays

**Fig. 2:**
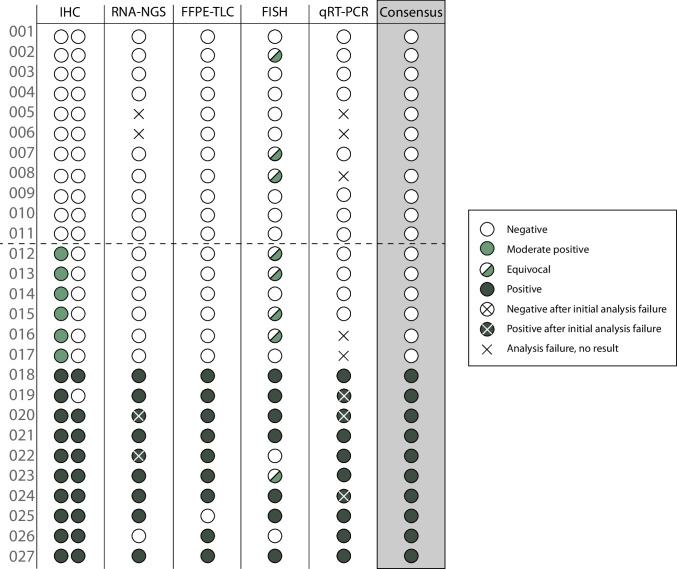
Comparison of *NTRK* fusion detection by IHC, RNA-NGS, FFPE-TLC, FISH, and qRT-PCR and the consensus result. For IHC, the assessment of both pathologists is shown. In case of discordancy between the pathologists, the highest intensity score was used underlining the use as screening method. The IHC of sample 019 is shown in Supplementary Fig. S2. Eleven pan-TRK IHC negative and 16 pan-TRK IHC positive tumors were analyzed. A total of 10 *NTRK* fusions were detected

**Fig. 3:**
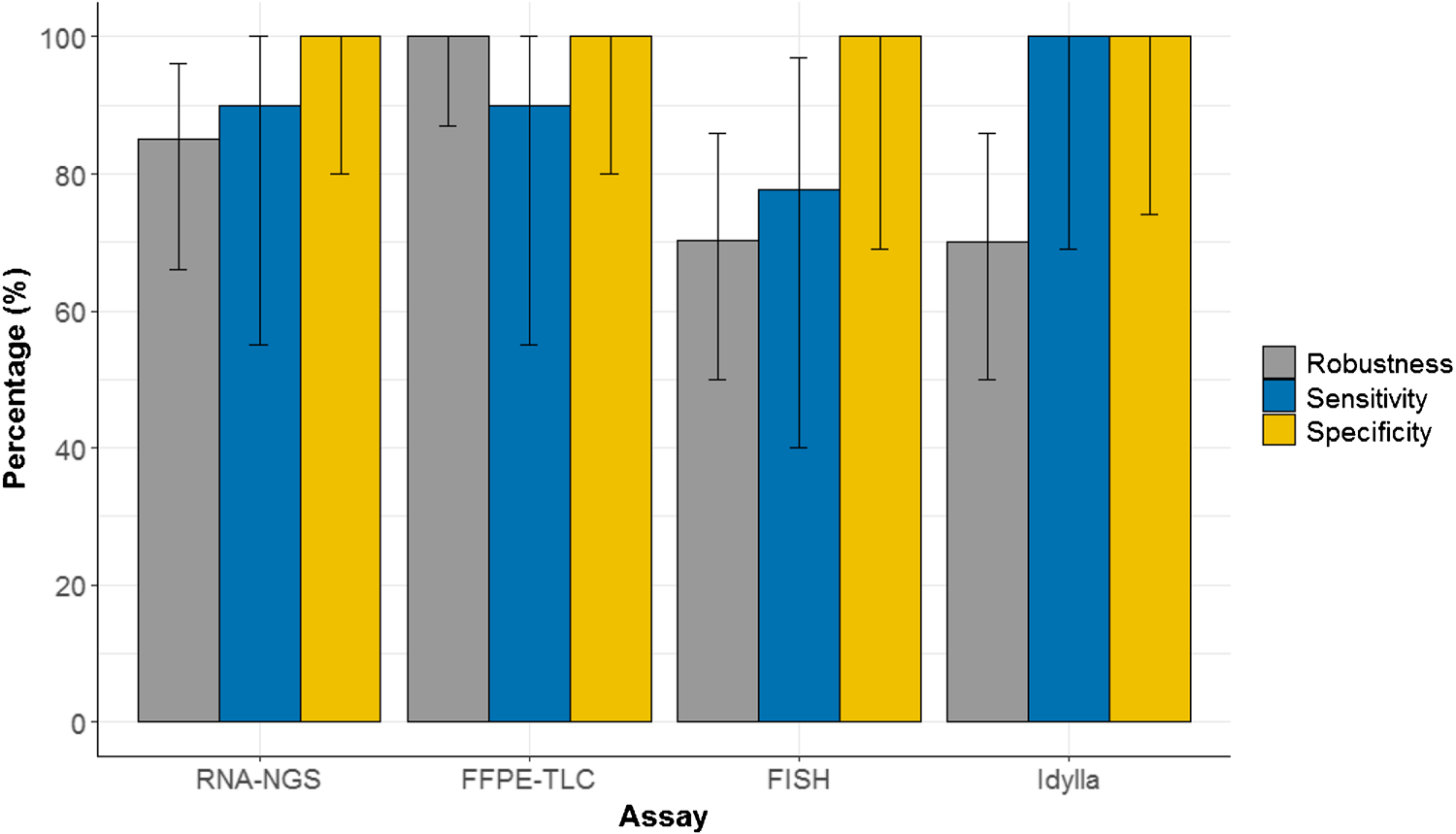
Robustness, sensitivity, and specificity of RNA-NGS, FFPE-TLC, FISH, and qRT-PCR as compared to the consensus result. Error bars represent 95% confidence interval

16 pan-TRK IHC positive and 11 negative tumor samples were included in the comparative analysis and analyzed with RNA-NGS, FFPE-TLC, FISH, and qRT-PCR (Fig. 2). Median age of the 27 FFPE tumor blocks that were analyzed was 43 months (range: 26–119 months), and median percentage of tumor cells in these tissues was 25% (range: < 10%–90%). For RNA-NGS, all samples passed the RNA quantity check but four samples did not generate an interpretable result. These samples had a median age of 65 months (range: 41–83 months) and contained a median tumor cell percentage of 40% (range: < 10%–90%) (Supplementary Table S3). One of these samples met the RNA quantity and quality check, but did not provide a result due to a high deduplication rate (119:1). The three other samples did not pass the RNA PCR quality test. For two out of four patients, a second FFPE tissue block was available and RNA-NGS was repeated successfully. Altogether, in 23 out of 27 samples, an interpretable result was generated in the first run, resulting in a robustness of 85% (95% CI: 66–96%) (Fig. 3). For FFPE-TLC, no repeat measurements were needed and robustness was 100% (95% CI: 87–100%). FISH analysis was successful for all tissue samples, but eight out of 27 samples were equivocal, resulting in a 70% (95% CI: 50–86%) robustness. For qRT-PCR, robustness was 70% (95% CI: 50–86%). Eight samples generated a non-interpretable result, including three technical errors related to the cartridge. Three of the invalid qRT-PCR samples also generated a non-interpretable result for RNA-NGS (Fig. 2). In three out of six samples, a valid result was generated after repeating the analysis, and two samples could not be repeated.

### *NTRK* fusion detection

An *NTRK* fusion was detected in 10 out of 16 (63%) pan-TRK IHC positive tumors according to the consensus results of all assays (Fig. 2). No *NTRK* fusions were detected in the 11 pan-TRK IHC negative samples with weak, non-specific staining. Nine tumors harbored an *NTRK1* fusion with different fusion partners (4 × *TPM3*, 4 × *LMNA*, 1 × *SFPQ*), one tumor harbored an *ETV6::NTRK3* fusion. All 10 tumor samples showed strong cytoplasmic TRK expression and nine of these patients also showed additional nuclear, perinuclear, or membranous TRK expression (Table 1).

**Table 1:** Results for pan-TRK immunohistochemistry, RNA-NGS, FFPE-TLC, FISH, and qRT-PCR for all confirmed *NTRK* fusions according to the consensus result

	IHC intensity	IHC staining pattern^*^	RNA-NGS	FFPE-TLC	FISH	qRT-PCR
018	Strong	C + P	*LMNA::NTRK1*	*LMNA::NTRK1*	*NTRK1* fusion	*NTRK1* fusion
019	Strong	C + N	*ETV6::NTRK3*	*ETV6::NTRK3*	*NTRK3* fusion	*NTRK3* fusion
020	Strong	C + P	*TPM3::NTRK1*	*TPM3::NTRK1*	*NTRK1* fusion	*NTRK1* fusion
021	Strong	C	*SFPQ::NTRK1*	*SFPQ::NTRK1*	*NTRK1* fusion	*NTRK1* fusion
022	Strong	C + P	*TPM3::NTRK1*	*TPM3::NTRK1*	Negative	*NTRK1* fusion
023	Strong	C + M	*TPM3::NTRK1*	*TPM3::NTRK1*	Equivocal	*NTRK1* fusion
024	Strong	C + P	*TPM3::NTRK1*	*TPM3::NTRK1*	*NTRK1* fusion	*NTRK1* fusion
025	Strong	C + M	*LMNA::NTRK1*	Negative	*NTRK1* fusion	*NTRK1* fusion
026	Strong	C + N	Negative^**^	*LMNA::NTRK1*	Negative	*NTRK1* fusion
027	Strong	C + M	*LMNA::NTRK1*	*LMNA::NTRK1*	*NTRK1* fusion	*NTRK1* fusion

*IHC*, = pan-TRK immunohistochemistry; *C*, = cytoplasmic; *P*, = perinuclear; *N*, = nuclear; *M*, = membranous

* In tumors with little tumor cell cytoplasm, the distinction between perinuclear or membranous staining could not reliably be made

** Re-analysis revealed a *LMNA::NTRK1* fusion

**Fig. 4:**
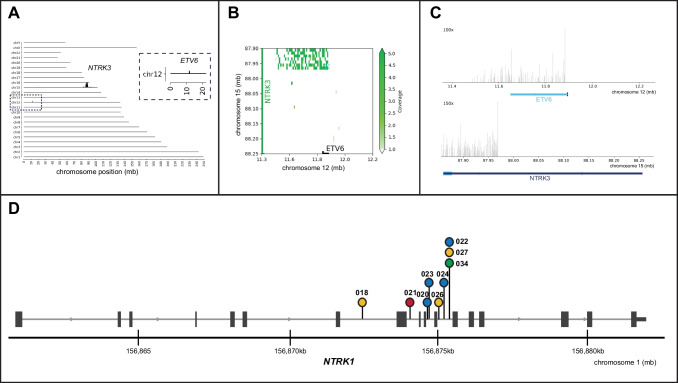
Overview of FFPE-TLC results. (**A**) Genome-wide coverage of proximity ligation fragments containing *NTRK3* sequences retrieved from sample 019. The rearranged region to the *NTRK3* gene is identified by the clustered fragments around the *ETV6* gene. (**B**) Butterfly plot confirming a true rearrangement within the targeted region. Proximity-ligation products between the target gene (*NTRK3*) and rearrangement partner (*ETV6*) are depicted in green. The transcriptional start site of both genes is indicated by the X mark. (**C**) Detailed overview of sequencing coverage on *NTRK3* and *ETV6*. (**D**) Lollipop overview of breakpoint positions identified in *NTRK1* and the corresponding fusion partner. The color of the dots indicates the 5′ fusion partner of *NTRK1*; *TPM3* (blue), *LMNA* (yellow), *SFPQ* (red), and *TPR* (green). Lollipop numbers correspond with sample numbers

RNA-NGS and FFPE-TLC detected nine *NTRK* fusions (Supplementary Table S4). Both assays missed a *LMNA::NTRK1* fusion (samples 025 and 026). Sample 026 showed strong pan-TRK staining including nuclear staining. We repeated the RNA-NGS using a different FFPE tissue block, even though the sample past the RNA quantity and quality check, as the high deduplication ratio (130:1) that was found in the first run might indicate limited RNA quality. The repeated RNA-NGS did reveal the *LMNA::NTRK1* fusion. The failure to detect the *LMNA::NTRK1* fusion in sample 025 using FFPE-TLC remains unclear. FFPE-TLC was able to automatically detect the interchromosomal fusion *ETV6::NTRK3* (Fig. 4A). The identified breakpoint position was in one of the large introns of *NTRK3* and predicted an in-frame fusion product (Fig. 4B and C). The other eight intrachromosomal *NTRK1* fusions had breakpoints between exons 8–12 and were detected after visual inspection (Fig. 4D).

Using FISH, three tumors met the criteria of ≥ 10% tumor cells with break apart signals, including the *ETV6::NTRK3* fusion. Furthermore, four *NTRK1* fusions were detected by single red signals only, including three *LMNA::NTRK1* fusions (Supplementary Table S5). Two *LMNA::NTRK1* fusions and a *TPM3::NTRK1* fusion were not detected by FISH.

All *NTRK* fusions were detected by qRT-PCR (Supplementary Table S6). Seven out of ten *NTRK* fusions were detected in the first run. The other three *NTRK* fusion-positive samples were detected after repeated qRT-PCR assays.

The overall agreement between the two molecular biologists for a consensus *NTRK* fusion score was 100% and is shown in the last column of Fig. 2. The sensitivity of RNA-NGS and FFPE-TLC was both 90% (95% CI: 55–100%). For qRT-PCR, sensitivity was 100% (95% CI: 69–100%). FISH had the lowest sensitivity: 78% (95% CI: 40–97%). In this comparative analysis of 27 samples, all tests showed 100% specificity set against the consensus decision.

### Sensitivity of pan-TRK IHC using FFPE-TLC

To validate the excellent sensitivity (100%) of pan-TRK IHC in the 11 pan-TRK negative samples (Fig. 2), we additionally performed FFPE-TLC in the high *NTRK* fusions prevalent (22%) subgroup of mCRC patients with a MSI-H/dMMR, *BRAF*, and *RAS* wild-type tumor. Only pan-TRK IHC negative samples were selected, in which FFPE-TLC could be performed in 22 out of 23 samples. A *TPR::NTRK1* fusion was detected by FFPE-TLC in sample 034 (Fig. 4D; Supplementary Fig. S3). This fusion was also detected by RNA-NGS in 100% of a small number of reads (*n* = 6) that showed intron fusions only, indicative that the fusion was not present in RNA reads, but only in DNA reads. The absence of RNA reads and the negative IHC suggests that the fusion was not transcribed. Therefore, it was concluded that FFPE-TLC identified a non-functional *TPR::NTRK1* fusion. Accordingly, pan-TRK IHC identified all functional *NTRK* gene fusions.

### Detection of other somatic alterations

A broader panel of genes was assessed, when performing RNA-NGS and FFPE-TLC for the detection of *NTRK* rearrangements (Supplementary Table S4). Concordance between RNA-NGS and FFPE-TLC was 100% for the detection of *BRAF*^*V600E*^ and *KRAS* mutations. All tumors harboring an *NTRK* fusion (*n* = 10) were *BRAF* and *KRAS* wildtype.qRT-PCR and RNA-NGS also assess rearrangements in *ALK*, *ROS1*, and *RET* and detect *MET* exon 14 skipping, but these alterations were not detected in the tumors analyzed in this study.

## Discussion

In this study, we show that FFPE-TLC and the qRT-PCR are reliable alternatives to the recommended RNA-NGS for the detection of *NTRK* fusions after IHC screening in mCRC, with a sensitivity of 90%, 100%, and 90%, respectively. Robustness for FFPE-TLC (100%) was good, reasonable for RNA-NGS (85%), but unsatisfactory for qRT-PCR (70%). Considering the high number of equivocal results and a limited sensitivity of 78%, we consider FISH analysis an inappropriate method for the detection of *NTRK* fusions in mCRC.

According to international guidelines, we used pan-TRK IHC as a screening tool to enrich for *NTRK* fusions [7, 8]. However, discriminating between weak, moderate, and strong TRK expression can be difficult and can lead to high inter-rater variability [3, 20]. To minimize variability, pan-TRK IHC was performed for all samples by the same optimized protocol, assessed by two experienced pathologists (Fig. 2, first column), and performed in a single cancer type (CRC). In this standardized and high-volume research setting, we found a 100% sensitivity and specificity for strong (3 +) TRK expression by IHC. However, in our opinion, this high sensitivity of pan-TRK IHC is unattainable in the routine diagnostic setting. This is reflected by the discordant pan-TRK IHC score in the *NTRK3* fusion sample (Supplementary Fig. S2). A lower sensitivity for *NTRK3* fusions by IHC has been described before [3]. Therefore, we recommend any above background positive pan-TRK IHC to be further analyzed by RNA or DNA based tests. For tumor (sub)types with a high prevalence of *NTRK* fusions, including MSI-H/dMMR, *BRAF* and *RAS* wildtype mCRC, upfront testing with RNA-NGS, FFPE-TLC, or qRT-PCR could be a reasonable approach.

Our results do not support FISH analysis for the detection of *NTRK* fusions in mCRC, which is in line with previous recommendations [7, 16]. Sensitivity of FISH was limited, possibly due to the occurrence of intrachromosomal *NTRK1* fusions, including *TPM3::NTRK1* and *LMNA::NTRK1*, which were most prevalent in our study. In addition, its usual advantages of being an inexpensive and easy assay are not apparent here, because three different break-apart probes are needed for *NTRK1*, *NTRK2*, and *NTRK3*, raising costs and complexity.

According to the results of this study, RNA-NGS, FFPE-TLC, and qRT-PCR can be used interchangeably in our opinion. Besides diagnostic performance, other factors are therefore important when deciding which assay to use, including costs, required input, turnaround time, availability, and whether or not simultaneous direct assessment of other somatic alterations is possible.qRT-PCR is an easy and fast assay to test for *NTRK* fusions with a turnaround time of only three hours and has a high diagnostic accuracy with an overall agreement of 100%, which is in line with previous reports [14, 17]. The most important limitation of qRT-PCR was the high rate of invalid test results. We found a robustness of 70% which is somewhat lower than reported in the aforementioned studies (88% and 89%, respectively). This may be explained by the use of older samples (median 43 months) with lower tumor percentages (median 25%) in our study. Increasing the tissue input seemed to increase the success rate.

RNA-NGS and FFPE-TLC are more expensive tests, but can be used more broadly by detecting other relevant mutations and rearrangements in one assay. In our study, RNA-NGS and FFPE-TLC performed equally well in terms of sensitivity. However, there are some important differences between these two assays. FFPE is the standard for preservation of tissue material in virtually all pathology labs worldwide. In FFPE tissue, DNA and RNA can be considerably damaged and fragmented, hindering DNA- and RNA-based NGS. For example, RNA-NGS could not detect an *NTRK* fusion in a tumor sample (case 026) that passed the RNA quality control. We hypothesized that the high deduplication ratio (130:1) was a sign of diminished RNA quality. Therefore, caution is advised for the interpretation of negative results in these instances. For FFPE-TLC, this fragmentation is irrelevant as FFPE-TLC is a proximity-ligation assay. On the other hand, transcription is not proven for fusions detected on DNA level by FFPE-TLC, emphasizing the importance of protein overexpression determined by IHC. Also, FFPE-TLC is not yet commercially available, so the perfect robustness needs to be confirmed in routine clinical practice. For broad implementation of FFPE-TLC, automated identification and characterization of rearrangements is needed. Therefore, further development of the computational pipeline PLIER is needed to automatically detect all intrachromosomal fusions. Nevertheless, the diagnostic performance and robustness could make FFPE-TLC a useful diagnostic tool for accurate detection of *NTRK* fusions.

This study provides a unique head-to-head comparison of available tests for the detection of *NTRK* fusions in a large population-based cohort of MSI-H/dMMR mCRC patients. Our study has several limitations. First, despite using this unbiased nationwide cohort, the number of detected *NTRK* fusions is low due to the rare occurrence of *NTRK* fusions in mCRC. Second, we used the consensus results as a reference standard with which we strived to find the ground truth. Because this reference standard is based on the assays that are being compared, sensitivity and specificity for these assays might be overestimated [21]. However, we think this epidemiological phenomenon might be limited in our study because two independent molecular biologists interpreted all results and were 100% concordant. Third, we did not perform a cost analysis which is an important factor for the adoption of an assay in daily practice. For determining total costs, not only materials and reagent cost should be taken into account but also hands-on-time costs, maintenance costs, and depreciation costs for all materials that are used.

We did not include whole genome sequencing (WGS) in our analysis because this is not feasible on FFPE material. However, WGS could be a solution for the identification of the diverse set of genetic biomarkers linked to the increasing number of targeted anticancer agents. Recently, the WIDE study has shown the feasibility of WGS for metastatic cancer in routine clinical practice [22]. However, costs are still very high, and the need for a repeat biopsy to obtain fresh-frozen tissue and the limited robustness (70%) are currently hurdles to overcome before WGS can be a suitable diagnostic tool in clinical practice.

To conclude, in this study, we compared pan-TRK IHC, RNA-NGS, FFPE-TLC, FISH, and qRT-PCR for the detection of *NTRK* fusions in dMMR/MSI mCRC, to find alternative diagnostic strategies for *NTRK* fusion detection that fit the need in routine care. We do not recommend FISH for screening or confirmation of *NTRK* fusions in mCRC due to its low sensitivity and robustness. Pan-TRK IHC is an appropriate screening tool for MSI-H/dMMR mCRC patients and *NTRK* fusions can be reliably confirmed by RNA-NGS, but also by FFPE-TLC and qRT-PCR.

## Supplementary Information

Below is the link to the electronic supplementary material.

Supplementary file 1 (DOCX 2638 KB)

Supplementary file 2 (XLSX 263 KB)

## Data Availability

All results of our analysis are provided in supplementary files. Other data obtained during the current study are available from the corresponding author on reasonable request.
