# Supplementary material for: Comparison of NTRK fusion detection methods in microsatellite-instability-high metastatic colorectal cancer
Source: Virchows Arch. 2023 Apr 17;482(6):983–92. doi: 10.1007/s00428-023-03538-1 (PMC10247849; doi:10.1007/s00428-023-03538-1)
Supplement: Supplementary file 1 — (DOCX 2638 KB) [file 428_2023_3538_MOESM1_ESM.docx]

**
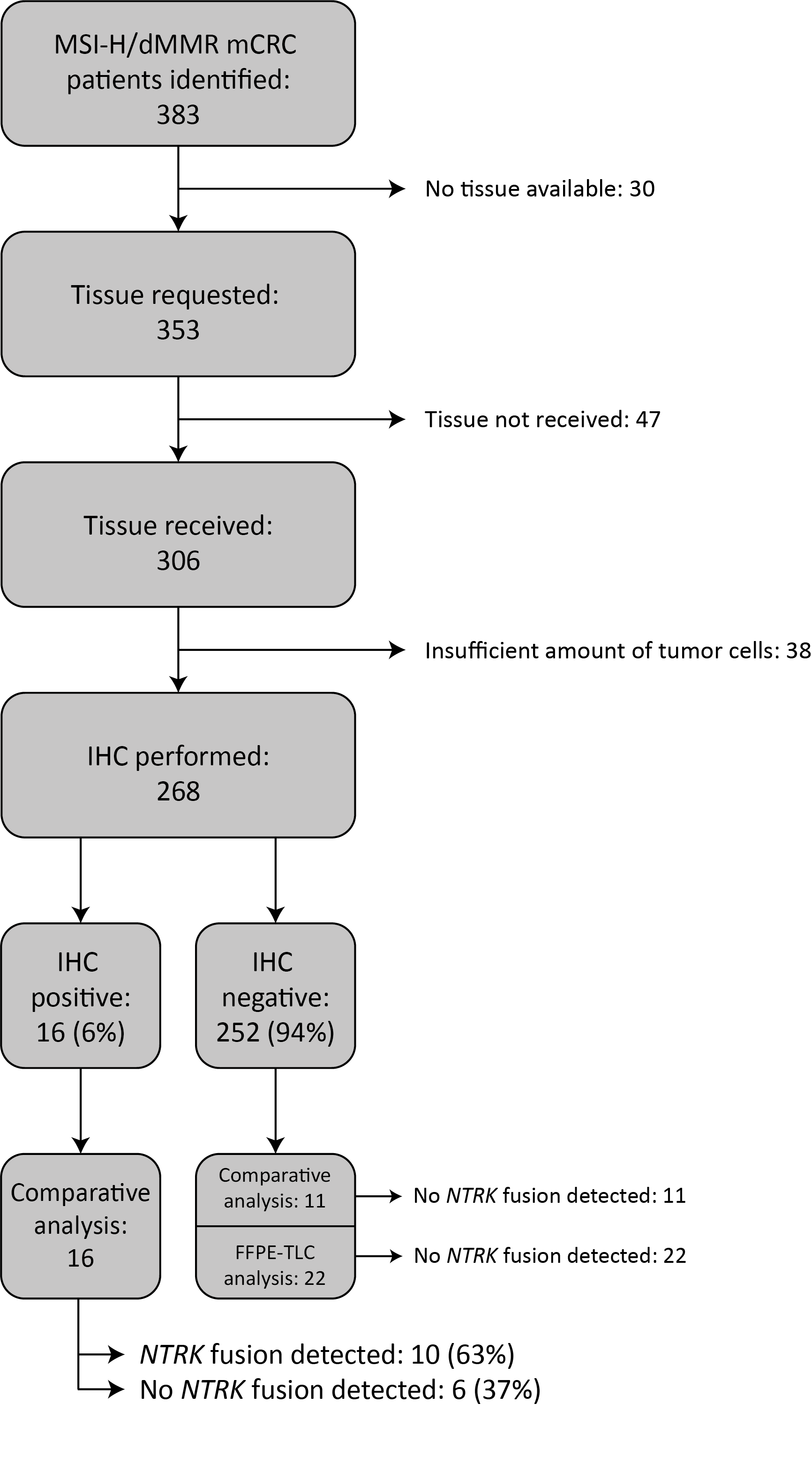
**

**Figure S1**: Study flow chart.
CRC = colorectal cancer; dMMR = deficient mismatch repair; IHC = immunohistochemistry; MSI-H = microsatellite-instability-high; FFPE-TLC = FFPE-Targeted Locus Capture.


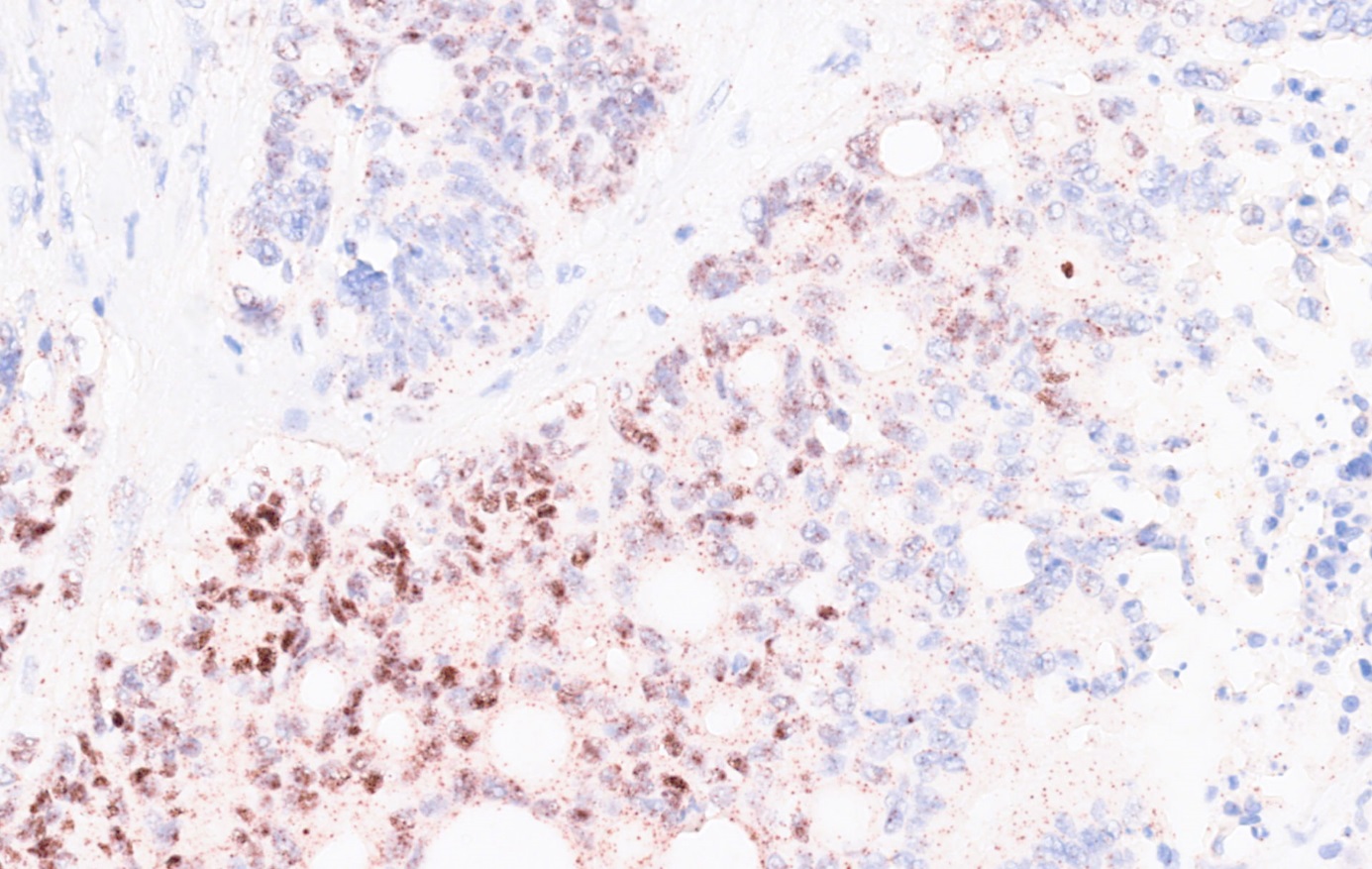


**Figure S2:** Pan-TRK immunohistochemistry of sample 019, 40x original magnification. The sample was scored negative by one pathologist and strongly positive with nuclear staining in 10% of tumor cells by the other pathologist. A *ETV6::NTRK3* fusion was detected by RNA-NGS, FFPE-TLC, FISH, and Idylla.


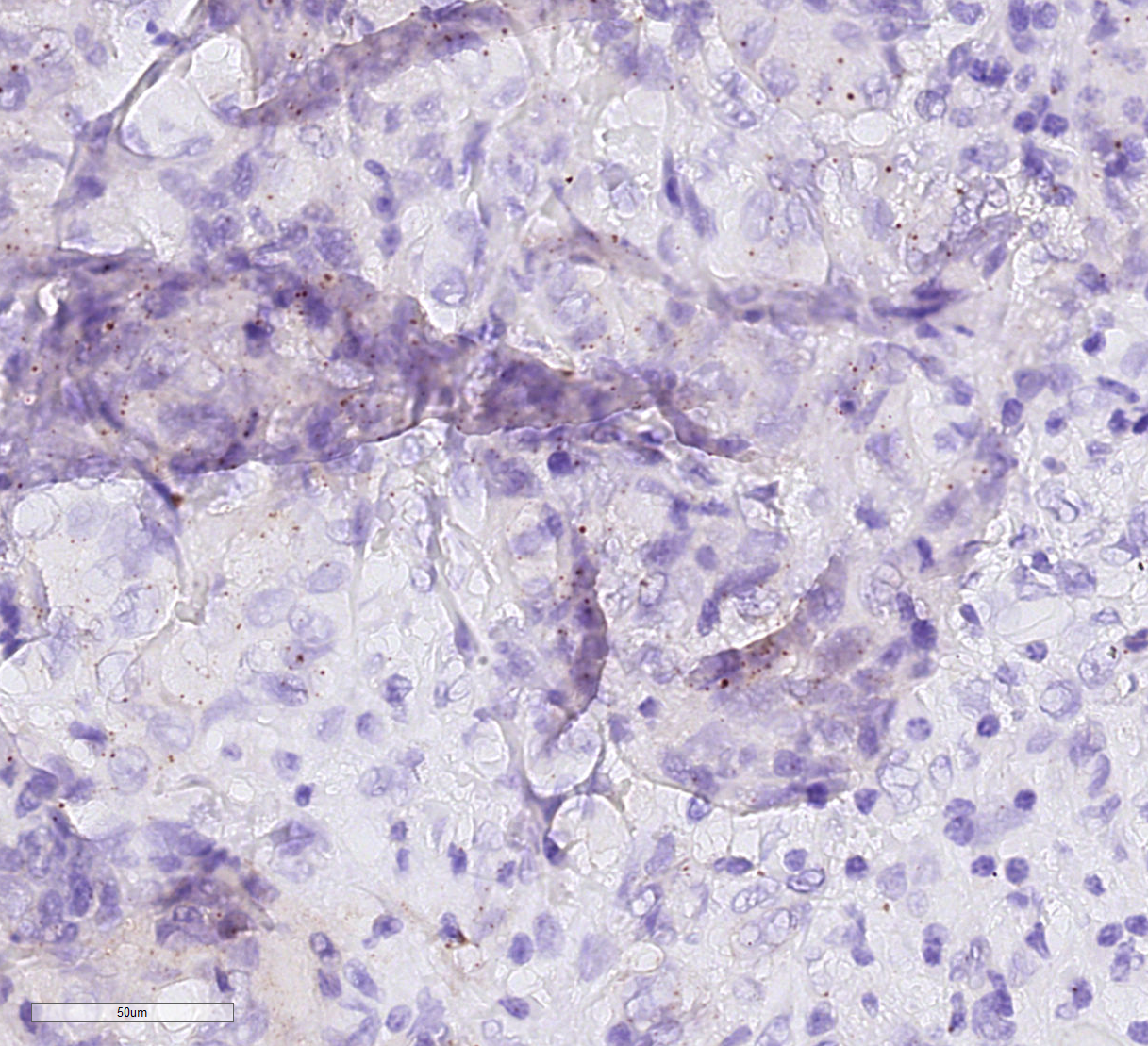


**Figure S3:** Pan-TRK immunohistochemistry without any positive staining, 40x original magnification. A *TPR::NTRK1* fusion was detected on DNA level by FFPE-TLC and RNA-NGS, but this fusion was not transcribed given the absence of RNA reads and the negative immunohistochemistry.
